# Increased susceptibility to otitis media in a *Splunc1*-deficient mouse model

**DOI:** 10.1242/dmm.019646

**Published:** 2015-05-01

**Authors:** Jennifer A. Bartlett, David K. Meyerholz, Christine L. Wohlford-Lenane, Paul W. Naumann, Nita H. Salzman, Paul B. McCray

**Affiliations:** ^1^ Department of Pediatrics, Carver College of Medicine, University of Iowa, Iowa City, IA 52242, USA; ^2^ Department of Pathology, Carver College of Medicine, University of Iowa, Iowa City, IA 52242, USA; ^3^ Department of Pediatrics, Division of Gastroenterology, Medical College of Wisconsin, Milwaukee, WI 53226, USA

**Keywords:** SPLUNC1, Otitis media, Mouse model, Middle ear, Eustachian tube, Host defense

## Abstract

Otitis media (inflammation of the middle ear) is one of the most common diseases of early childhood. Susceptibility to otitis is influenced by a number of factors, including the actions of innate immune molecules secreted by the epithelia lining the nasopharynx, middle ear and Eustachian tube. The SPLUNC1 (short palate, lung, nasal epithelial clone 1) protein is a highly abundant secretory product of the mammalian nasal, oral and respiratory mucosa that is thought to play a multifunctional role in host defense. In this study we investigated Splunc1 expression in the ear of the mouse, and examined whether this protein contributes to overall host defense in the middle ear and/or Eustachian tube. We found that Splunc1 is highly expressed in both the surface epithelium and in submucosal glands in these regions in wild-type mice. In mice lacking Splunc1, we noted histologically an increased frequency of otitis media, characterized by the accumulation of leukocytes (neutrophils with scattered macrophages), proteinaceous fluid and mucus in the middle ear lumens. Furthermore, many of these mice had extensive remodeling of the middle ear wall, suggesting a chronic course of disease. From these observations, we conclude that loss of Splunc1 predisposes mice to the development of otitis media. The *Splunc1^−/−^* mouse model should help investigators to better understand both the biological role of Splunc1 as well as host defense mechanisms in the middle ear.

## Abstract (precis)

**Summary:** We document expression of the innate immune factor Splunc1 in the murine middle ear and Eustachian tube, and describe spontaneous development of otitis media in mice lacking functional Splunc1.

## INTRODUCTION

Otitis media, or inflammation of the middle ear, is one of the most familiar illnesses of early childhood. By the age of 3, over 80% of children have had at least one episode of otitis media (Teele et al., 1989), making it one of the most common reasons for physician visits and accounting for an estimated US$2.88 billion to US$3.8 billion in direct and indirect healthcare costs each year in the United States (O'Brien et al., 2009; Ahmed et al., 2014). The inflammation associated with otitis media can be infectious or non-infectious. Although acute otitis media often resolves with antibiotic treatment, the condition can progress to prolonged or recurrent forms known as chronic otitis media with effusion or chronic suppurative otitis media. Over time, chronic otitis media can lead to complications such as conductive hearing loss (Teele et al., 1990; Klausen et al., 2000), and problems with balance and motor coordination (Casselbrant et al., 1995; Gawron et al., 2004). Otitis media is a multifactorial condition, with a variety of environmental risk factors including daycare outside the home, parental smoking, the presence of siblings and lack of breastfeeding (Uhari et al., 1996). Studies with twins and triplets indicate that there is also a significant genetic component to otitis susceptibility (Casselbrant et al., 2004). 

To defend the middle ear from environmental insults, the epithelia of the nasopharynx, middle ear and Eustachian tube secrete an array of antimicrobial and immune modulatory molecules. In the chinchilla, these secretions include the proposed innate immune protein Splunc1 (short palate, lung, nasal epithelial clone 1; also known as PLUNC, LUNX, NASG, SPURT and BPIFA1) (McGillivary and Bakaletz, 2010). In addition to its expression in the ear, Splunc1 expression is widespread throughout the conducting airways. In humans and other mammals, Splunc1 is expressed by the surface epithelium and submucosal glands and ducts of the trachea, bronchus, nasal epithelium, nasopharynx and salivary glands (Weston et al., 1999; Bingle and Bingle, 2000; LeClair et al., 2001; Sung et al., 2002; Wheeler et al., 2002; Di et al., 2003; Campos et al., 2004; Bingle et al., 2005; Larsen et al., 2005; Kim et al., 2006; Vargas et al., 2008; McGillivary and Bakaletz, 2010; Musa et al., 2012). Accordingly, the protein is present in human nasal secretions (Ghafouri et al., 2002, 2003b, 2004; Casado et al., 2005; Kim et al., 2006), tracheal aspirates (Campos et al., 2004) and saliva (Ghafouri et al., 2003a), as well as in porcine bronchoalveolar lavage (Bartlett et al., 2013). The emerging picture suggests that Splunc1 is a highly abundant secretory product of the mammalian respiratory system, whose expression extends from the upper respiratory tract into the oral cavity, nasopharynx and middle ear.

Although the precise biological function of the Splunc1 protein is not yet well-defined, there is growing evidence that it participates in host defense in the conducting airways. In humans, altered SPLUNC1 levels are associated with exposure to allergens (Seshadri et al., 2012), airway irritants (Fornander et al., 2013), and chronic bacterial colonization and airway inflammation due to cystic fibrosis (Bingle et al., 2007). Animal studies suggest that Splunc1 has antibacterial and/or immunomodulatory effects. Although *Splunc1*-deficient mice do not develop spontaneous lung disease in the absence of a bacterial insult, they do exhibit impaired responses to intrapulmonary challenge with *Mycoplasma pneumoniae* (Gally et al., 2011), *Pseudomonas aeruginosa* (Liu et al., 2013a) and *Klebsiella pneumoniae* (Liu et al., 2013b), and overexpression of Splunc1 protects mice from inhaled *P. aeruginosa* (Lukinskiene et al., 2011). Although direct microbicidal action is the mechanism most frequently invoked to explain this Splunc1-mediated protective effect, Splunc1 is also proposed to possess immunomodulatory (Thaikoottathil et al., 2012), anti-biofilm (Gakhar et al., 2010) and/or chemotactic (Sayeed et al., 2013) properties.

**TRANSLATIONAL IMPACT**

**Clinical issue**

Otitis media, or inflammation of the middle ear due to infectious or non-infectious causes, is a very frequent medical problem in infants and young children. The economic impact of this problem is substantial because it affects the majority of children at some point in their lives and often necessitates doctor visits. Susceptibility to otitis media is influenced by a complex interplay of environmental and genetic risk factors, some of which have been explored using knockout mouse models. In many of these models, otitis arises spontaneously owing to anatomical defects, such as craniofacial malformations that impair Eustachian tube function or cilia in the middle ear and/or Eustachian tube. Less is known, however, about how loss of innate immune factors might also predispose to the development of middle ear disease.

**Results**

Here, we describe spontaneous development of otitis media in mice lacking the innate immune molecule Splunc1 (short palate, lung, nasal epithelial clone 1). Splunc1 is best known as a highly expressed secretory product of the conducting airways, where it participates in host defense against a variety of airway pathogens. We found that, in addition to its expression in airway tissues, Splunc1 is also expressed by the epithelia of the middle ear and Eustachian tube in the mouse. Our histopathological analysis indicates that *Splunc1^–/–^* mice develop otitis media at a higher frequency than their wild-type littermates, with an increased incidence of inflammatory markers such as leukocytes and cell debris in the middle ear lumen. We also observed remodeling of the middle ear epithelium, suggesting that otitis media is a chronic problem in these mice.

**Implications and future directions**

The *Splunc1^–/–^* mouse represents a new mouse model with which to study the complex biological origins of otitis media. The Splunc1 protein is thought to play a multifunctional role in host defense, with proposed antibacterial, anti-biofilm, immunomodulatory and chemotactic properties. Therefore, our findings suggest the interesting possibility that loss of Splunc1 function might render *Splunc1^–/–^* mice vulnerable to otitis through multiple overlapping mechanisms. Additionally, the insights provided by studies of the *Splunc1^–/–^* mouse have potential therapeutic implications because this study points to the *SPLUNC1* gene as a possible contributor to otitis media susceptibility in the human population and might suggest possibilities for therapeutic interventions to treat or prevent this illness.

Given its distribution throughout the conducting airways, most studies of *Splunc1^−/−^* mice have centered on identifying defects in lung host defense. The lack of spontaneous lung disease in *Splunc1^−/−^* mice suggests that this protein plays a host defense role that is redundant with other immune molecules found in the airways, or that its function is most relevant in specific contexts (e.g. early infection). In this study, we examined the consequences of Splunc1 deficiency in tissues outside the respiratory tract, focusing on the middle ear and Eustachian tube. In contrast to the lung phenotype in these animals, we discovered spontaneous middle ear disease in the *Splunc1^−/−^* mice. Here, we characterize Splunc1 expression in the murine middle ear and Eustachian tube, and investigate the incidence of otitis media in *Splunc1^−/−^* animals.

## RESULTS

**Fig. 1.**
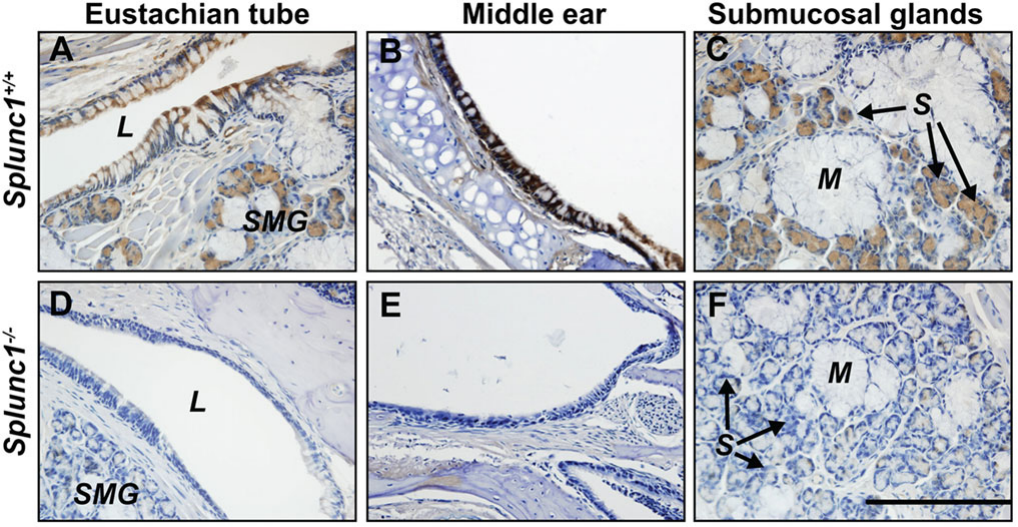
**Splunc1 protein is expressed in the mouse middle ear and Eustachian tube.** Splunc1 (brown stain) is present in the ciliated and non-ciliated cells of the surface epithelium lining the Eustachian tube (A) and middle ear (B) in the mouse. Splunc1 is also abundant in the serous cells of the submucosal glands in these tissues (A,C). Splunc1 expression is completely absent in the middle ears and Eustachian tubes of *Splunc1^−/−^* mice (D-F). L, lumen of the Eustachian tube; SMG, submucosal glands; M, mucous tubules; S, serous acini. Scale bar: 200 µm.

Splunc1 mRNA and protein is reportedly expressed in the middle ear and Eustachian tube of the chinchilla (McGillivary and Bakaletz, 2010). Using immunohistochemistry, we observed a very similar expression pattern for Splunc1 in the wild-type mouse. In these animals, Splunc1 protein is abundantly expressed in both the middle ears and Eustachian tubes (Fig. 1), with a pattern very similar to that in the conducting airways. In the Eustachian tube, which possesses a pseudostratified epithelium continuous with the respiratory epithelium of the nasopharynx, Splunc1 expression localizes to ciliated and nonciliated columnar epithelial cells (Fig. 1A). A similar distribution was observed for the surface epithelium of the middle ear (Fig. 1B). Additionally, substantial Splunc1 expression was noted in serous cells of the submucosal glands throughout both the middle ear and Eustachian tube regions (Fig. 1B,C). Splunc1 protein was completely absent from these tissues in *Splunc1^−/−^* mice (Fig. 1D-F).

Considering the abundance of Splunc1 in these regions in wild-type mice, we hypothesized that loss of Splunc1 would increase susceptibility to infection and/or inflammation in the middle ear. To explore this hypothesis, we performed a histological analysis on head sections from a total of 26 *Splunc1^−/−^* mice and 18 age-matched *Splunc1^+/^*^+^ littermate controls to look for evidence of acute and/or chronic otitis. Reasoning that the characteristic changes to the epithelium associated with recurrent infection and/or inflammation would be most apparent in older mice, we focused on relatively aged mice (10-18 months of age; median age for both groups was 10 months).

**Fig. 2.**
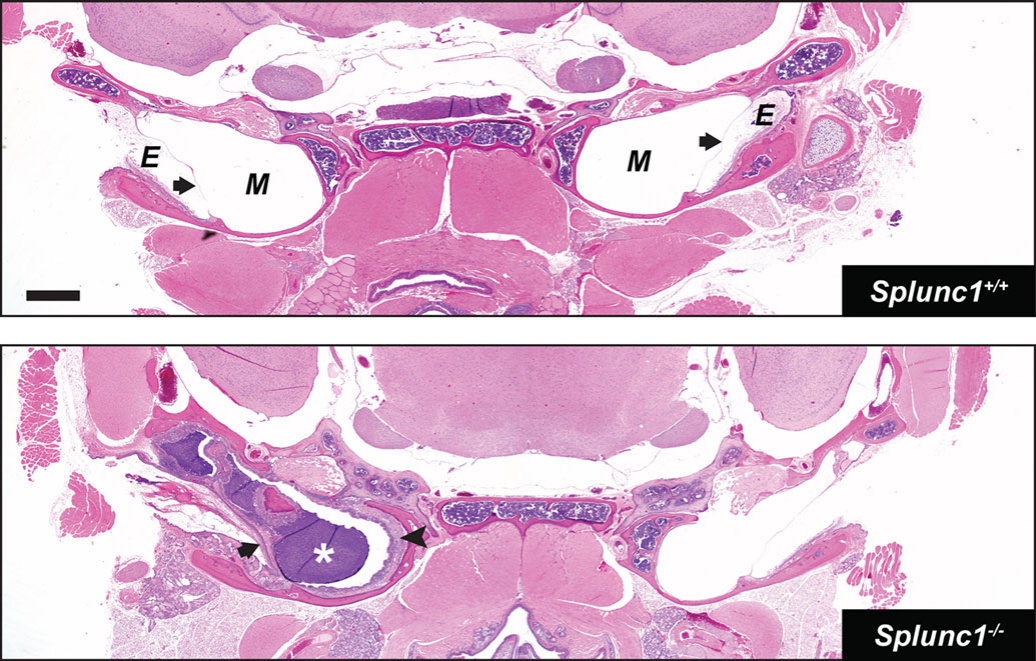
**Unilateral otitis media in a *Splunc1^−/−^* mouse.** H&E-stained coronal sections through the heads of 10- to 18-month-old *Splunc1*^+/+^ and *Splunc1^−/−^* mice were inspected for gross abnormalities. The top panel depicts a representative coronal section from a *Splunc1^+/+^* mouse. The bottom panel is from a *Splunc1^−/−^* mouse exhibiting unilateral otitis media, characterized by purulent material in the middle ear lumen (white asterisk). In this mouse, thickening of the tympanic membrane and middle ear epithelium (black arrowhead) are indicative of chronic otitis media. E, external ear canal; M, middle ear; in both panels, black arrows indicate the tympanic membranes. Scale bar: 0.7 mm.

**Fig. 3.**
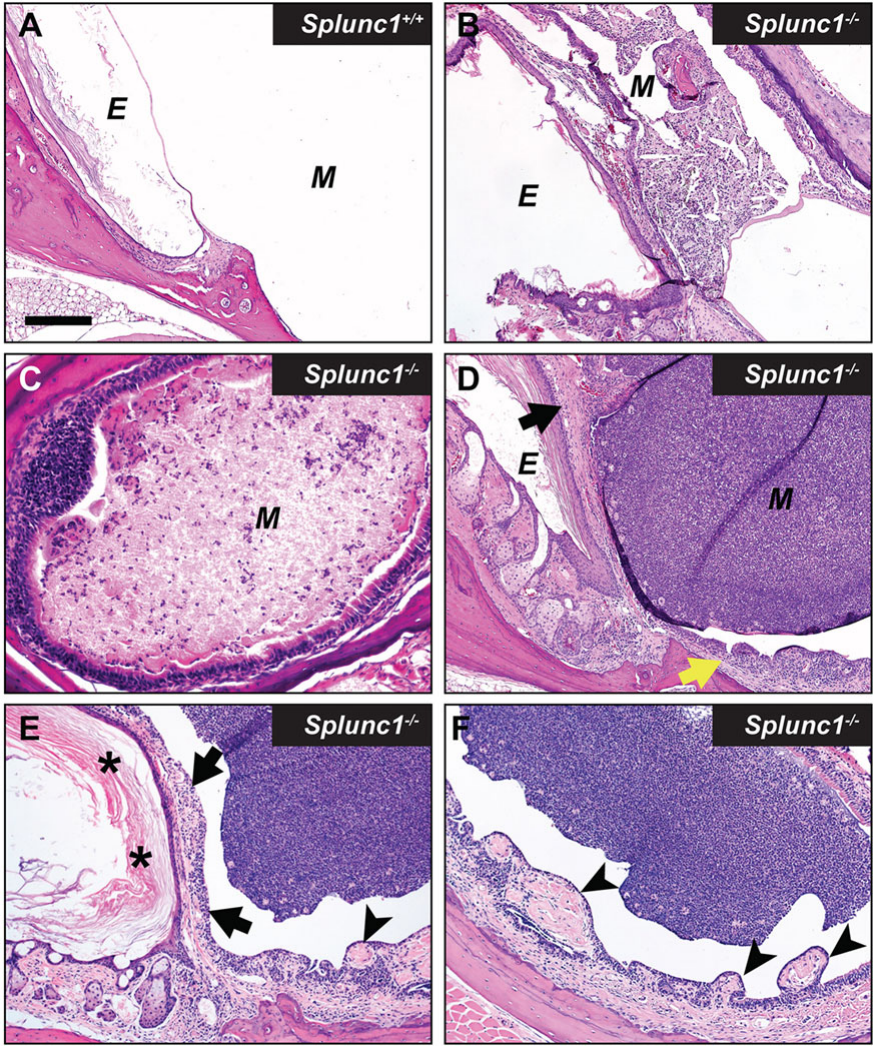
**Otitis media in *Splunc1^−/−^* mice is incompletely penetrant.** Panel A depicts the lumen of the middle ear from a representative *Splunc1^+/+^* mouse, which is largely free of PMNs or epithelial hyperplasia. In contrast, middle ears from different *Splunc1^−/−^* mice (B-F) exhibited variable severity with respect to inflammation and epithelial remodeling at the time of necropsy. The abundance of accumulated PMNs and cell debris in the middle ear lumens of these mice ranged from moderate (B,C) to very significant (D-F). Occasionally, small spicule-shaped spaces known as cholesterol clefts (indicative of cholesterol crystals in the original tissue) were observed along with cellular debris in the middle ears of *Splunc1^−/−^* mice (B). Some *Splunc1^−/−^* mice also presented with varying amounts of epithelial proliferation and mucosal thickening in the middle ear (D, yellow arrow) and tympanic membrane (D, black arrow), suggesting a history of repeated otitis. In the mouse depicted in panels E and F, remodeling manifests as thickening of the tympanic membrane (black arrows) by hyperkeratosis (asterisks), inflammation and increased connective tissue. The region of middle ear adjacent to inflammation often had increased connective tissue, epithelial proliferation and/or polyploid changes (arrowheads). E, external ear canal; M, middle ear. Scale bar: 180 µm (A,B,D-F); 90 µm (C).

**Fig. 4.**
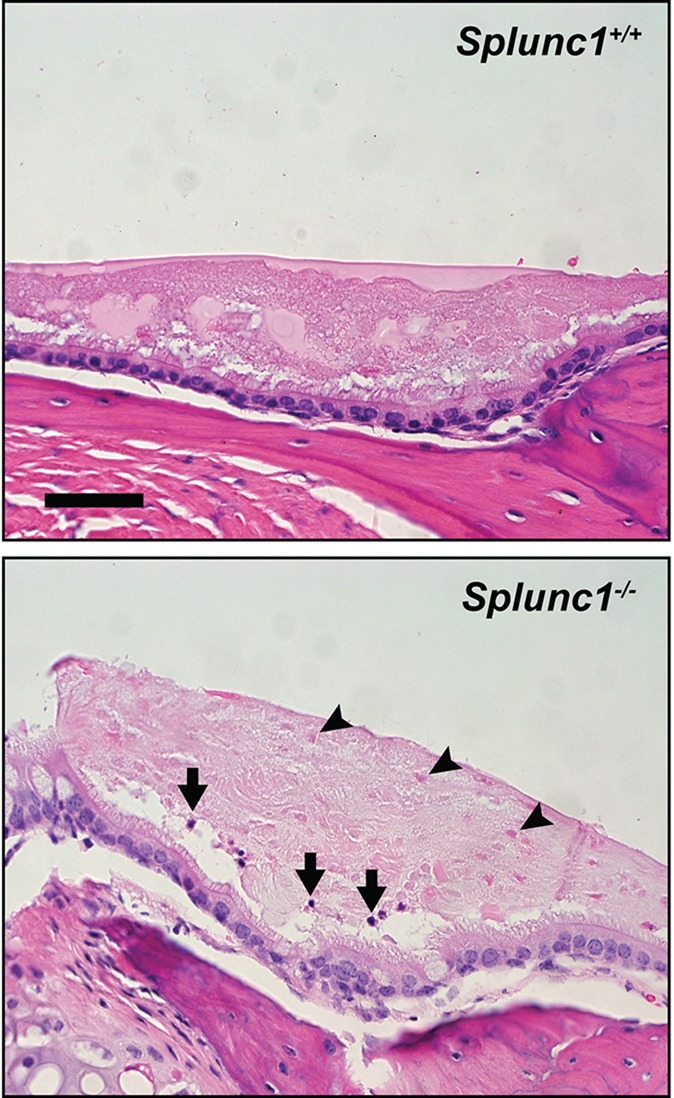
**The middle ears of *Splunc1^−/−^* mice harbor increased inflammatory cells and cellular debris relative to wild-type** **littermate controls.** The top panel depicts a representative image of a middle ear from a *Splunc1^+/+^* mouse, in which the middle ear epithelium is covered by a layer of fluid containing globular fluid material. In the *Splunc1^−/−^* middle ear image (bottom panel) this fluid contains multiple punctate eosinophilic ‘ghost’ cells (black arrowheads) along with a small number of solitary PMNs (black arrows). Scale bar: 43 µm.

We found that, as a group, *Splunc1^−/−^* mice exhibited an increased incidence of otitis relative to the wild-type control mice at the time of necropsy. This phenotype was incompletely penetrant, with *Splunc1^−/−^* mice exhibiting a range of severity with respect to inflammatory markers and epithelial changes. Several *Splunc1^−/−^* mice (8 out of 26; 30.8%) exhibited overt otitis media, associated with mucopurulent material completely or partially filling the middle ear lumen (Figs 2 and 3). Otitis was generally unilateral (Fig. 2), although a bilateral presentation was observed in one case. In contrast, overt otitis was noted in only 1 out of 18 (5.5%) wild-type mice. In *Splunc1^−/−^* mice with more moderate phenotypes, modest numbers of polymorphonuclear neutrophils (PMNs) could be seen within the middle ear lumen (Figs 3 and 4).

Interestingly, our studies suggest that many of the *Splunc1^−/−^* mice had likely experienced recurrent episodes of otitis. Histological findings indicative of chronic otitis media included frequent remodeling of the middle ear epithelium, characterized by epithelial hyperplasia, polyps and thickening of the middle ear mucosa (Figs 2 and 3). Less frequently, we observed cholesterol clefts admixed in cellular debris in the middle ear lumen (Fig. 3B). We additionally noted that, relative to their wild-type counterparts, *Splunc1^−/−^* mice tended to exhibit greater amounts of cell debris in the fluid of the middle ear, including eosinophilic ‘ghost’ cells (Fig. 4). Although the origin of these ghost cells is not well-established, we speculate that they represent dead PMNs possibly associated with past inflammatory events, because ghost cells and scattered PMN infiltration were sometimes seen in conjunction.

**Fig. 5.**
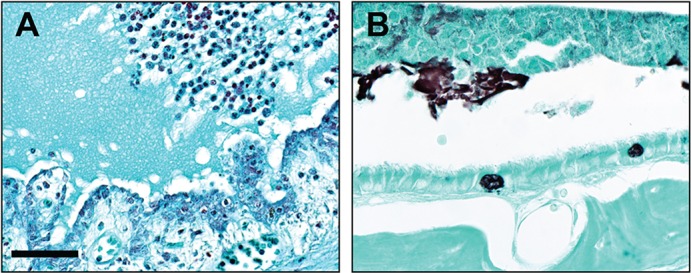
**Lack of evidence for bacterial or fungal organisms in *Splunc1^−/−^* middle ears.** Middle ear sections from *Splunc1^−/−^* mice with otitis were examined for the presence of microorganisms. (A) A modified Gram stain was negative for bacteria. (B) Gomori methenamine silver (GMS) staining was performed to survey for fungal organisms. In this representative image, GMS staining produced typical black staining of both goblet cell mucus and luminal secreted mucus, but was negative for fungal organisms in luminal material. Scale bar: 57 µm (A); 38 µm (B).

The inciting event for the observed inflammation in *Splunc1^−/−^* middle ears was unclear because histological stains of middle ear sections from affected mice were negative for bacterial and fungal pathogens (Fig. 5). When histological methods failed to uncover evidence for microbial infections in these middle ears, we used fluorescence *in situ* hybridization (FISH) to search for bacterial genetic material in tissue sections from affected mice. Middle ear sections from *Splunc1^−/−^* mice exhibiting otitis, as well as unaffected wild-type controls, were hybridized with a ‘universal’ bacterial probe recognizing a conserved region of the 16S rRNA (Amann et al., 1990). This approach also failed to detect bacteria in the middle ears of the mice (not shown).

**Fig. 6.**
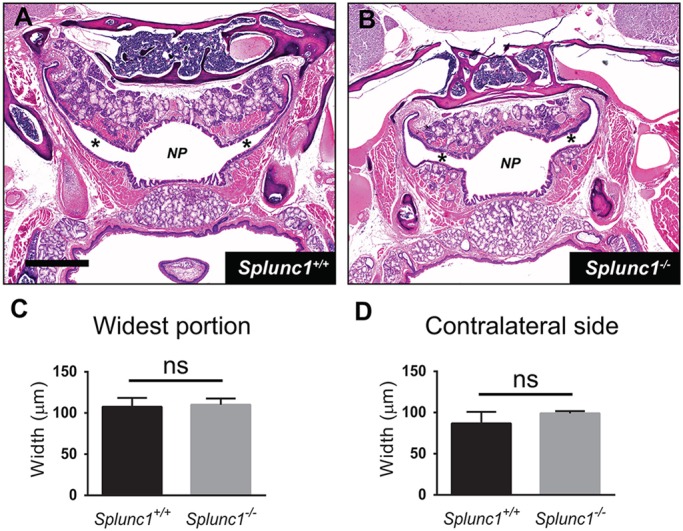
**Assessment of Eustachian tube patency in *Splunc1^+/+^* and *Splunc1^−/−^* mice.** The nasopharynx and Eustachian tubes were visualized in coronal sections from *Splunc1^+/+^* and *Splunc1^−/−^* mice (representative images in panels A and B, respectively). NP, nasopharynx; asterisks indicate the lumens of Eustachian tubes. Scale bar: 1.1 mm. To assess patency at the time of necropsy, Eustachian tubes were measured to determine width at their widest point (summarized in C), as well as at their widest point on the contralateral side (D). In these graphs, bars depict the mean and s.e.m. for *n*=4 *Splunc1^+/+^* and *n*=4 *Splunc1^−/−^* mice. Unpaired *t*-tests were performed to test for statistically significant differences.

We also looked for pathology of the Eustachian tube in the *Splunc1^−/−^* mice. A common contributing factor in otitis media is loss of patency in the Eustachian tube, which can prevent drainage and clearance of pathogens from the middle ear. To help maintain patency, the Eustachian tube epithelium normally secretes surfactant molecules that lower the opening pressure between the middle ear and the nasopharynx. Of note, Splunc1 has previously been demonstrated to exhibit surfactant-like properties (Gakhar et al., 2010). Therefore, we hypothesized that loss of *Splunc1* expression might result in persistent loss of Eustachian tube patency in *Splunc1^−/−^* mice. To address this, we examined Eustachian tubes in coronal head sections from *Splunc1^+/+^* and *Splunc1^−/−^* mice (aged 11-17 months) to assess the frequency of Eustachian tube collapse at the time of necropsy. As shown in Fig. 6, we were unable to observe evidence for collapsed Eustachian tubes in either *Splunc1^+/+^* or *Splunc1^−/−^* mice (Fig. 6A,B). Furthermore, average Eustachian tube width measurements were similar between the wild-type and *Splunc1^−/−^* mice (Fig. 6C,D).

**Fig. 7.**
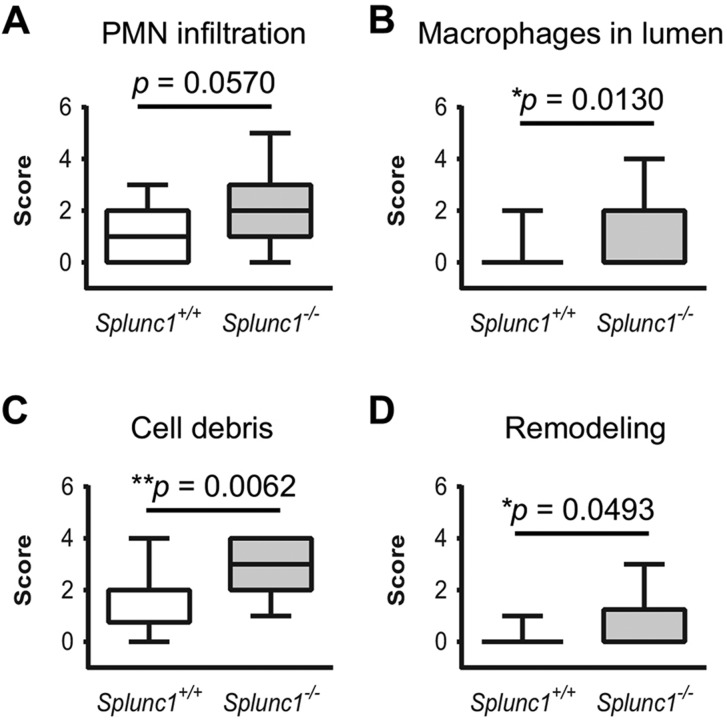
**Summary of histopathological analysis of *Splunc1^−/−^* and wild-type mouse middle ears.** Middle ear sections from *Splunc1^−/−^* mice and age-matched wild-type littermate controls were examined for histological evidence of otitis or other significant changes in the middle ear, using scoring rubrics described in the Materials and Methods. Sections were scored for several parameters including the infiltration of PMNs and macrophages in the middle ear lumen (panels A and B, respectively), accumulated cell debris (C), and the degree of hyperplasia and remodeling exhibited by the epithelium (D). Scores are summarized as box and whiskers plots. In these plots, the median score is denoted by a thick line, the box indicates the 25th and 75th percentiles, and the minimum and maximum values are depicted by the outer bars. Mann–Whitney tests were used to test for statistically significant differences (*n*=18 *Splunc1^+/+^* and *n*=26 *Splunc1^−/−^* mice).

To compare the overall frequency of otitis media in the *Splunc1^−/−^* mice and controls, a Fisher exact test was performed for ‘overt otitis’ in the two populations. Using this approach, we observed a trend toward increased otitis frequency in the *Splunc1^−/−^* mice that did not quite reach statistical significance (*P*=0.0603). In support of this, histological scoring indicated a trend toward increased PMN recruitment to the middle ear lumen, accompanied by significantly greater numbers of macrophages and increased cell debris and remodeling in the middle ears of *Splunc1^−/−^* mice relative to wild-type controls (summarized in Fig. 7).

## DISCUSSION

Our findings indicate that Splunc1 is highly expressed in the epithelium and glandular tissue of the middle ear and Eustachian tube of the mouse, and that loss of Splunc1 from these regions predisposes animals to develop otitis media. Thus, we can add the *Splunc1^−/−^* mouse to the list of models that feature spontaneous chronic otitis media as a component of their phenotype. A review of other otitis-prone mouse models highlights the diverse biological processes that can influence susceptibility to this condition. In the eyes absent homolog 4 (*Eya4*^−/−^) mouse and the Tail-short (Ts) mouse (which harbors a deletion in the ribosomal-subunit gene *Rpl38*), craniofacial and Eustachian tube dysmorphologies result in abnormal clearance and/or drainage of the middle ear and Eustachian tube (Depreux et al., 2008; Noben-Trauth and Latoche, 2011). Mutations leading to ciliary defects (such as those seen in the *Chibby-* and *Spag6*-knockout mice) also predispose to otitis, owing to reduced mucociliary clearance in these regions (Voronina et al., 2009; Li et al., 2014). Chronic otitis is also associated with loss of the transcription factors *Fbxo11* (mutated in the *Jeff* mouse model), *Evi1* (deleted in the *Junbo* mouse model) and transforming growth factor 1 (*Tgif*), which affect a broad range of developmental and other phenomena through their involvement in the TGF-β signaling pathway (Hardisty-Hughes et al., 2006; Parkinson et al., 2006; Tateossian et al., 2013). Susceptibility to otitis might also be impacted by deficiencies in innate immune molecules, such as the microbial pattern recognition receptors Toll-like receptor 4 (TLR4) (MacArthur et al., 2006) and Toll-like receptor 2 (TLR2) (Leichtle et al., 2009), and the immune signaling molecule MyD88 (Hernandez et al., 2008).

We have yet to determine how loss of Splunc1 leads to increased otitis susceptibility. However, the multifunctional nature of this protein suggests that there could be multiple mechanisms contributing to the *Splunc1^−/−^* middle ear phenotype. There is some evidence to support the notion that *Splunc1^−/−^* mice are predisposed to develop otitis media owing to loss of antibacterial activity by the Splunc1 protein. Several groups have reported that Splunc1 has direct bactericidal and/or bacteriostatic effects (Chu et al., 2007; Zhou et al., 2008; Sayeed et al., 2013), although it seems that these effects are limited to certain pathogens. Of interest, McGillivary and colleagues demonstrated that Splunc1 protein inhibited the growth *in vitro* of nontypeable *Haemophilus influenzae* (NTHi), one of the organisms most frequently associated with otitis media (McGillivary and Bakaletz, 2010). However, *in vivo* experiments suggested that *Splunc1* knockdown in the middle ear of the chinchilla did not significantly impair the animal's ability to handle an acute NTHi challenge, making these findings somewhat difficult to interpret (McGillivary and Bakaletz, 2010).

Previous reports indicate that, as a surfactant, Splunc1 inhibits bacterial growth indirectly by interfering with biofilm formation (Gakhar et al., 2010; Liu et al., 2013b). Biofilms contribute to the persistence of otitis-causing microbes in the middle ear (Post, 2001; Ehrlich et al., 2002; Hall-Stoodley et al., 2006; Hoa et al., 2009; Thornton et al., 2011); it is possible that loss of Splunc1 results in overgrowth of bacterial biofilms and a reduced ability to clear bacteria from the ear. It is important to point out that we were unable to find evidence for bacteria in the middle ears of the *Splunc1^−/−^* mice, by histological methods or by looking for bacterial genetic material, which suggests that we might be observing a sterile inflammation process in the *Splunc1^−/−^* middle ear. As such, our findings might reflect a necessity for Splunc1 in maintaining homeostasis in the middle ear space, with loss of Splunc1 resulting in increased cell death and debris and a consequent influx of inflammatory cells. Additionally, we cannot rule out the possibility that the *Splunc1^−/−^* middle ears could have harbored bacteria at various time points throughout the lifespans of the mice, and that the inflammation seen in aged mice could represent responses to infections that failed to resolve normally.

The SPLUNC1 protein is thought to share evolutionary origins with lipopolysaccharide-binding protein (LBP) and bactericidal/permeability increasing protein (BPI). LBP and BPI both act as sensors for the bacterial cell-wall component lipopolysaccharide (LPS), coordinately regulating the presentation of LPS to its receptor, TLR4, and thus directing TLR4-mediated inflammatory responses. The evolutionary relationship between SPLUNC1 and both LBP and BPI has long invited speculation that SPLUNC1 might have either pro- or anti-inflammatory effects in the context of a bacterial infection or other inflammatory stimulus. It has been reported that SPLUNC1 binds LPS (Ghafouri et al., 2004; Sayeed et al., 2013), and SPLUNC1 binds directly to *P. aeruginosa* (Sayeed et al., 2013), suggesting that, like LBP and BPI, SPLUNC1 might act as a bacterial sensor molecule. Interestingly, mice lacking Tlr4 also spontaneously develop chronic otitis media (MacArthur et al., 2006), owing to their inability to mount a sufficient inflammatory response to bacterial pathogens; the observation of a similar phenotype in the *Splunc1^−/−^* mouse suggests that these mice suffer from a deficiency in Tlr4-mediated inflammation owing to altered responsiveness to LPS. It has also been observed that Tlr2 signaling in response to *M. pneumoniae*-derived lipoproteins is inhibited in the presence of Splunc1, possibly because Splunc1 directly binds the lipoproteins and prevents them from engaging the receptor Tlr2 (Chu et al., 2007).

In addition to possible roles in modulation of Tlr4- and Tlr2-mediated inflammation, Splunc1 has also been implicated in regulating allergic inflammation (Thaikoottathil et al., 2012) and in the response to a ‘sterile’ inflammatory stimulus, carbon nanotubes (Di et al., 2013). One measure of the acute inflammatory response – influx of PMNs and alveolar macrophages – was enhanced in the lungs of *Splunc1*-overexpressing mice after receiving carbon nanotubes (Di et al., 2013). This observation suggests that Splunc1 might have chemotactic properties, a finding that was confirmed directly in *in vitro* cell migration assays (Sayeed et al., 2013). Thus, a reduced capacity for leukocyte recruitment could also contribute to dysregulated inflammation in the *Splunc1^−/−^* middle ear.

A final consideration is the possibility of Eustachian tube dysfunction in the *Splunc1^−/−^* mouse. As described in the Results, we found no histological evidence for an increased frequency of Eustachian tube collapse in the *Splunc1^−/−^* mice. Although this most likely indicates that the Eustachian tube is not affected by loss of Splunc1 surfactant activity, it should be noted that analysis of histological sections might not be the most effective approach for capturing a dynamic process such as the opening and closing of the Eustachian tube. It is also possible that loss of Splunc1 surfactant activity reduces mucociliary clearance in the middle ears and Eustachian tubes of the *Splunc1^−/−^* mice. Several studies have documented the ability of surfactants to enhance mucociliary transport rates (Allegra et al., 1985; Kakuta et al., 1991; Ballard et al., 2006), and it has been proposed that one role of the Splunc1 protein could be to promote mucociliary clearance throughout the conducting airways (Gakhar et al., 2010; McGillivary and Bakaletz, 2010). McGillivary and Bakaletz (2010) measured reduced mucociliary clearance rates in the chinchilla Eustachian tube subsequent to *Splunc1* knockdown, supporting the idea that reduced clearance of bacteria and other inflammatory stimuli from the middle ear might contribute to middle ear infections in *Splunc1^−/−^* mice.

In conclusion, our results indicate that the phenotype of the *Splunc1^−/−^* mouse should now be broadened to include increased risk of spontaneous development of middle ear disease. Future studies with this animal model will likely include a more detailed analysis of the molecular processes that lead from *Splunc1* deficiency to the development of otitis. In addition to providing additional insight into the unique, and possibly varied, roles that the Splunc1 protein plays in mucosal host defense, this mouse should also serve as a useful model with which to explore the biological pathways that influence otitis susceptibility. In particular, these results suggest that genetic variation at the *SPLUNC1* locus could impact vulnerability to otitis media in the human population, and that measurement of SPLUNC1 levels in the middle ear might have predictive value in identifying patients at increased risk of developing chronic otitis. Along these lines, we note that Saferali and colleagues recently reported a single-nucleotide polymorphism (SNP; rs1078761) that is associated with a decreased abundance of SPLUNC1 protein, and worsened lung function, in the airways of individuals with cystic fibrosis (Saferali et al., 2015). These results support a role for SPLUNC1 in mucosal host defense, and imply that this protein might have therapeutic potential in the treatment and/or prevention of otitis media.

## MATERIALS AND METHODS

### Animals

This study was approved by the University of Iowa Animal Care and Use Committee (IACUC). All mice were housed under specific pathogen-free conditions, in accordance with the specifications of the National Institutes of Health. *Splunc1^−/−^* mice used in this study harbored a nonsense mutation in exon 50 of the *Splunc1* gene (L50X) (Liu et al., 2013b), on the C3HeB/FeJ strain background. This mutation is associated with complete ablation of *Splunc1* expression in the lungs. Heterozygous (*Splunc1^+/−^*) breeder mice were crossed to generate *Splunc1^−/−^* mice and *Splunc1^+/+^* littermate controls for all experiments. For this analysis, all mice were necropsied at 10-18 months of age.

### Tissue processing

Mice were euthanized by carbon dioxide exposure with confirmation of euthanasia by bilateral thoracotomy according to IACUC approved guidelines, and tissues immediately harvested. Skulls were dissected free of soft tissues, the lower jaw and brain removed, and skulls were placed in 10% neutral buffered formalin for 4 days. Following fixation, skulls were washed with tap water for 2 h and decalcified in 14% EDTA (Sigma-Aldrich, St Louis, MO, USA), pH 7.3, for 7 days with agitation. The skulls were washed for 2-3 h with tap water, placed back into 10% neutral buffered formalin and then routinely processed, paraffin embedded, and sectioned to view the middle ears or Eustachian tubes. Tissue sections were then either processed for immunohistochemical analysis or stained with hematoxylin and eosin (H&E) for histopathology. Additional stains used to survey for microorganisms included modified Gram staining (Stoltz et al., 2010) and Gomori methenamine silver (GMS) staining (Grocott, 1955).

### Immunohistochemistry

Head sections (6 µm) were heated in a 55°C oven for 15 min and deparaffinized by incubating with xylenes for two consecutive 15-min intervals. Sections were then washed twice with 100% ethanol (2 min per wash), followed by incubations for 2 min each in 95% ethanol, 75% ethanol, 50% ethanol, and double-distilled water. Antigen retrieval was achieved by incubating with Antigen Unmasking Solution (Vector Laboratories, Burlingame, CA, USA), diluted 1:100 in water and heated in a microwave. Endogenous peroxidase activity was quenched by washing sections twice with PBS and incubating for 30 min with a 0.3% H_2_O_2_ solution. The M.O.M.™ (‘Mouse on mouse’) Immunodetection Kit (Vector Laboratories) was used to detect Splunc1 protein expression in mouse head sections. To do this, sections were blocked for 1 h using the M.O.M.™ Mouse Ig Blocking reagent, washed twice with PBS, and incubated in the M.O.M.™ Diluent. Sections were then incubated overnight at 4°C with SPLUNC1 monoclonal antibody (R&D Systems, Minneapolis, MN, USA; catalog number MAB1897), diluted 1:1500 in M.O.M.™ Diluent. After washing with PBS, sections were incubated with M.O.M.™ Biotinylated Anti-Mouse IgG Reagent for 10 min at room temperature. Sections were washed again with PBS, followed by a 30-min incubation with VECTASTAIN^®^ ABC Reagent (Vector Laboratories). Following another PBS wash, color detection was performed by incubating sections with Vector^®^ DAB peroxidase substrate solution (Vector Laboratories). Sections were counterstained with hematoxylin and mounted in Permount mounting medium (Thermo Fisher Scientific, Waltham, MA, USA).

### Fluorescence *in situ* hybridization (FISH)

Mouse heads were fixed, processed and paraffin embedded as described above. Head sections (3 µm) were mounted on slides and FISH was performed as described by Canny et al. (2006). Briefly, slides were deparaffinized, dried, and hybridized with the Texas-red-labeled probe (Bact338 TR-GCTGCCTCCGTAGGAGT) (Operon Technologies, Huntsville, AL, USA) for 90 min at 50°C in hybridization buffer (0.9 M NaCl, 20 mM Tris-HCl pH 7.4, 0.05% SDS). Slides were washed for 5 min at 50°C in wash buffer (0.9 M NaCl, 20 mM Tris-HCl pH 7.4, 0.01% SDS), rinsed in water and allowed to air dry. Tissue sections were mounted with coverslips using Vectashield mounting medium (Vector Laboratories, Burlingame, CA, USA) for fluorescence. Slides were viewed by fluorescence microscopy using a Nikon E400 upright microscope. Images were captured using a Photometrics CoolSnap ES CCD camera (Photometrics, USA), and analyzed using Metaview software (Universal Imaging Corporation, Molecular Devices, USA). For these studies, mouse intestinal tissue served as a positive control for the probe.

### Histological analysis

Histological analysis was performed according to principles described by Gibson-Corley et al. (2013). H&E-stained head sections were examined by a pathologist masked to groups and scored as one batch for signs of infection and inflammation. In this analysis, ‘overt otitis’ refers to readily observable disease (inflammation/remodeling) that is apparent at low magnification (20× magnification) at histological examination. To assess the degree of PMN infiltration into the middle ear lumen, each middle ear section was scored according to the following ordinal scale: 0=no PMN in lumen; 1=1-5 PMN, isolated or scattered infiltrates in middle ear; 2=6-20 PMN, scattered or in very small loose aggregates in middle ear; 3=PMN cellular aggregates filling 25% of the middle ear; 4=PMN cellular aggregates filling 26-50% of the middle ear; 5=PMN cellular aggregates filling >50% of the middle ear. Middle ear lumens were scored for the presence of macrophages as follows: 0=no macrophages in lumen; 1=1-5 macrophages, isolated or scattered in middle ear; 2=6-20 macrophages, scattered or in very small loose aggregates in middle ear; 3=21-40 macrophages, cellular aggregates; 4=>40 macrophages, aggregates. Cellular debris in the middle ear was assessed according to this scale: 0=no debris in middle ear; 1=1-5, isolated or scattered in middle ear; 2=6-20, scattered or in very loose aggregates in middle ear; 3=21-40, cellular aggregates; 4=>40, aggregates. Epithelial remodeling was scored as follows: 0=normal; 1=attenuation of epithelium; 2=focal hyperplasia; 3=widespread epithelial hyperplasia and thickening, expansion of lamina propria, inflammatory cell exocytosis.

For all parameters, scores are presented as box and whisker plots representing a total of 18 *Splunc1^+/+^* and 26 *Splunc1^−/−^* mice. Mann–Whitney tests were performed to test for statistically significant differences between *Splunc1^+/+^* and *Splunc1^−/−^* mice for each parameter, assuming an α=0.05. Reported *P*-values represent two-tailed values in each case. The overall incidence of overt otitis media in the *Splunc1^+/+^* and *Splunc1^−/−^* groups was compared using a Fisher exact test, in which ‘overt otitis’ was defined as a score of 3 or greater in the PMN scale described above. Using this criterion, there was a total of 1 out of 18 wild-type mice and 8 out of 26 *Splunc1^−/−^* mice that were considered to exhibit overt otitis.

Eustachian tube width measurements were made using ImageJ, and were plotted as bar graphs representing the mean and s.e.m. For this data set, unpaired *t*-tests were performed to test for statistically significant differences, with an α=0.05. All statistical analyses were performed using GraphPad Prism, version 6.02.
